# Chemically engineered essential oils prepared through thiocyanation under solvent-free conditions: chemical and bioactivity alteration

**DOI:** 10.1007/s13659-024-00456-w

**Published:** 2024-06-01

**Authors:** Liz E. Lescano, Mario O. Salazar, Ricardo L. E. Furlan

**Affiliations:** 1 Farmacognosia, Facultad de Ciencias Bioquímicas y Farmacéuticas, Universidad Nacional de Rosario (UNR), Suipacha 531, 2000 Rosario, Argentina; 2 Consejo Nacional de Investigaciones Científicas y Técnicas (CONICET), Suipacha 531, 2000 Rosario, Argentina

**Keywords:** Chemically modified essential oils, Ammonium thiocyanate, Iodine catalysis, Bioactive compounds, Acetylcholinesterase inhibitors

## Abstract

The generation of chemically engineered essential oils (CEEOs) prepared from bi-heteroatomic reactions using ammonium thiocyanate as a source of bioactive compounds is described. The impact of the reaction on the chemical composition of the mixtures was qualitatively demonstrated through GC–MS, utilizing univariate and multivariate analysis. The reaction transformed most of the components in the natural mixtures, thereby expanding the chemical diversity of the mixtures. Changes in inhibition properties between natural and CEEOs were demonstrated through acetylcholinesterase TLC autography, resulting in a threefold increase in the number of positive events due to the modification process. The chemically engineered *Origanum vulgare* L. essential oil was subjected to bioguided fractionation, leading to the discovery of four new active compounds with similar or higher potency than eserine against the enzyme. The results suggest that the directed chemical transformation of essential oils can be a valuable strategy for discovering new acetylcholinesterase (AChE) inhibitors.

**Graphical Abstract:**

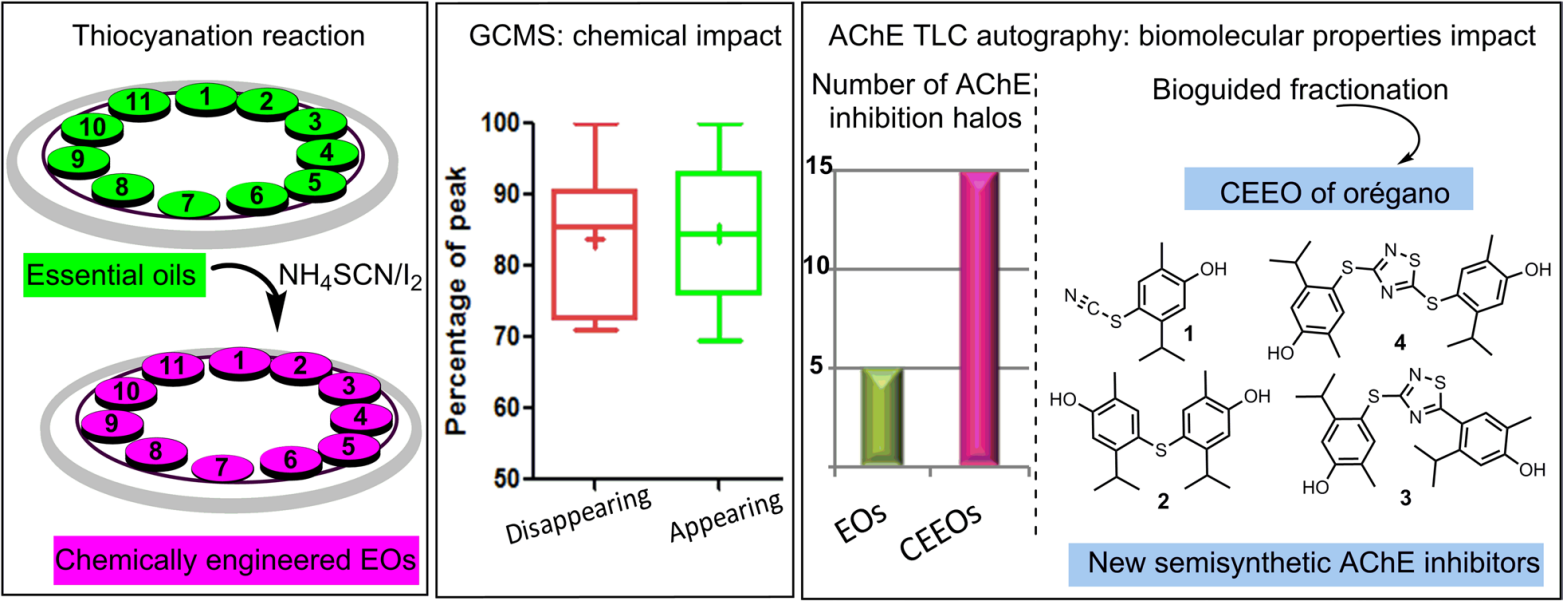

**Supplementary Information:**

The online version contains supplementary material available at 10.1007/s13659-024-00456-w.

## Introduction

Natural products (NPs) arise through the evolution of biosynthetic pathways, driven by their functional benefits to the host organism [1]. Consequently, NPs have become a prolific source of active compounds and primary chemical structures for discovering new drugs [2, 3]. Essential oils (EOs) are complex blends that may contain over 300 different natural volatile compounds [4, 5]. Generally, EOs consist of low molecular weight lipophilic hydrocarbons (both aromatic and aliphatic) and their oxygenated derivatives. EOs primarily serve vital functions in plant chemoecology [6] and also hold potential as a novel source of interesting lead structures for drug discovery [7].

The success of drug discovery is closely linked to the accessibility to libraries containing molecules with interesting biomolecular properties. Leveraging the fact that NPs have evolved to efficiently bind to biological macromolecules, various techniques have been proposed as approaches to enhance their chemical diversity [3, 8, 9], leading to the discovery of active compounds [10–14]. Nearly two decades after its appearance, the chemical diversification of extracts has become a complementary strategy in the search for bioactive molecules [15]. This methodology aims to transform as many components of natural extracts as possible through simple chemical reactions. Modifying a substantial number of components within an inactive natural extract could potentially generate one or more active compounds. To increase the chances of creating new compounds during the modification, it is beneficial to introduce functional groups that are uncommon or rarely found in nature [16].

Various reactions have been developed to produce chemically engineered extracts (CEEs) enriched with specific elements [17] such as nitrogen [15, 18], sulfur [19–21], bromine [22, 23], or fluorine [24]. All these reactions so far are referred to as mono-heteratomic, as they primarily increase the proportion of functionalities based on a single element of interest. These mono-heteratomic transformations have led to the discovery to products with increased biological activity [16, 18, 20, 21, 25–43]. These remarkable outcomes suggest a vast potential to further explore this strategy [44].

A comparison of heteroatom content in and NPs drugs has revealed that drugs contain significantly more sulfur and nitrogen that NPs [2, 45]. The frequency of NPs with functionalities based on both heteroatoms is very rare [46, 47].

Thiocyanate is a versatile synthon which has been used extensively in synthetic organic chemistry and contains many biological features that are useful in various sectors, such as medicine, pesticides, and materials [48, 49]. Organic chemists have concentrated their efforts into exploring thiocyanation reactions and applications, which mostly involve nucleophilic substitution favored by oxidants [48]. As an alternative, thiocyanate salts in combination with different oxidants have been used as the thiocyanate source. Among the thiocyanate salts, NH_4_SCN is one of the most reactive nucleophilic reagents available and is safe, nontoxic, inexpensive, stable, and easy to handle [50]. This salt is generally directly used to access alkyl thiocyanates, while their combination with an oxidant is required for the preparation of aromatic counterparts via electrophilic reactions or cross-coupling reactions [51, 52].

This salt can introduce the thiocyanate synthon by reaction with ketones possessing α-hydrogens [49] and (hetero)aromatic C-H bonds [50]. The carbonyl and aromatic groups are among the most common functional groups found in NPs [46, 47]. Therefore, the use of reactions that transform both of them may result attractive to alter the composition of natural extracts in a significant way. To explore the value of CEEs as a source of bioactive compounds, we present a method to modify EOs using NH_4_SCN as a derivatizing reagent. Unlike previous approaches, this reaction is the first capable of introducing sulfur and nitrogen in a single step. In this work, we use various tools to analyze how this reaction affects the chemical composition and certain biomolecular properties of the mixtures to a significant extent.

## Results and discussion

### Ammonium thiocyanate as the reagent and chemical impact evaluation

Most conventional chemical reactions frequently employ considerable quantities of volatile and toxic organic solvents, which give rise to substantial concerns regarding worker health and safety, as well as environmental pollution [53, 54]. Thus, the development of green, sustainable, efficient reactions in the absence of hazardous organic solvents is a desirable step and the design of solvent-free procedures to generate CEEOs. Taking advantage of the liquid nature of EOs, eleven commercial mixtures were modified by solvent-free reaction conditions using NH_4_SCN (derivative reagent) and iodine (oxidant) [55, 56].

The key to success with CEEs is producing a high number of compounds to maximize the chance of finding a bioactive one. In view of the extensive chemical diversity of NPs, it is imperative to employ analytical methodologies capable of detecting most of the components present in these mixtures. GC–MS is a widely used technique for the analysis of complex mixtures of natural origin, demonstrating its reliability in creating EOs fingerprints [6] and identifying hundreds of metabolites within these mixtures [57].

**Fig. 1:**
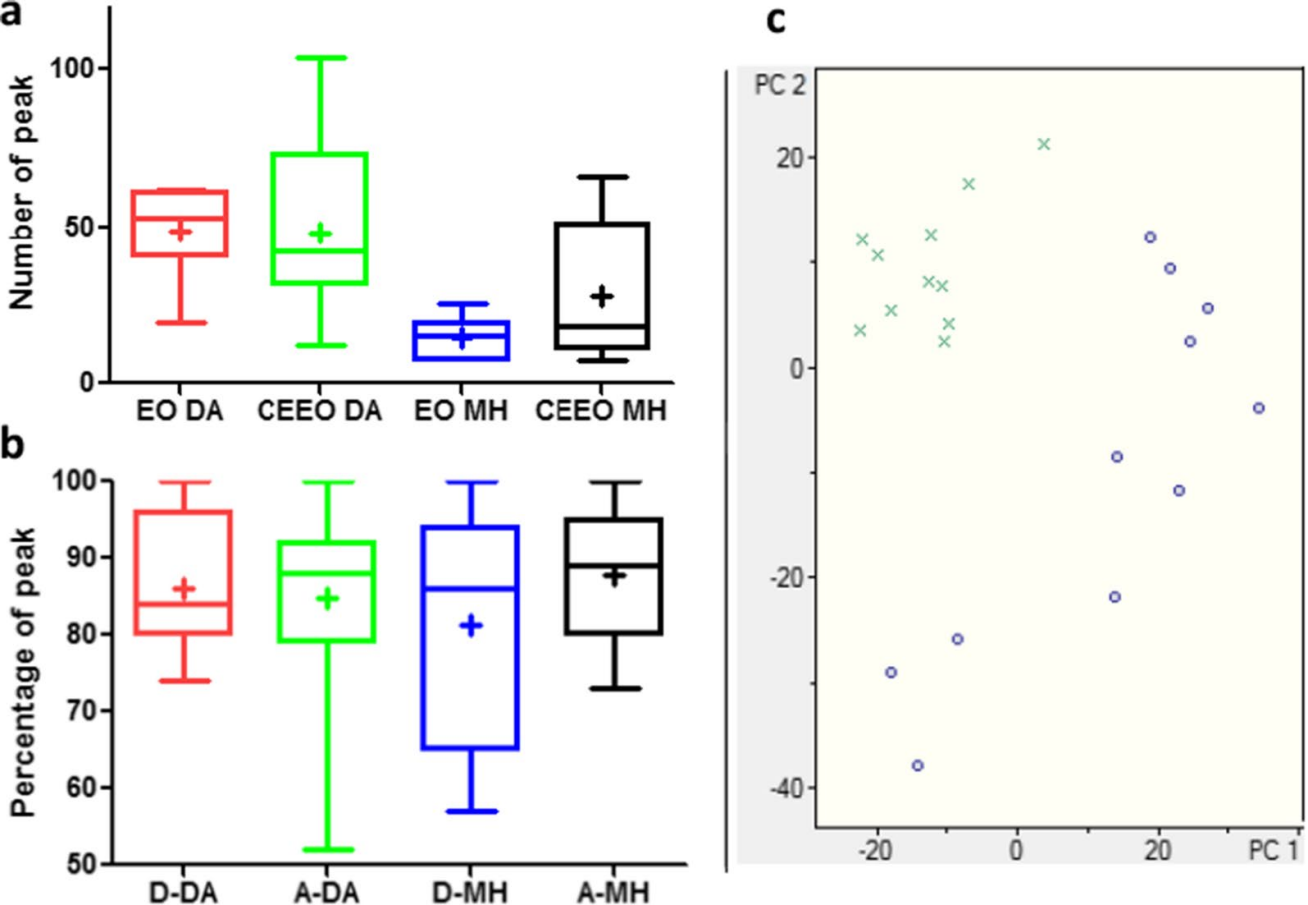
Box and whiskers plot for **a** number of peaks from EOs with DA and MH (red and blue respectively) and CEEOs with DA and MH (green and black respectively) detected in the GC–MS chromatograms, **b** percentage of peaks that disappeared with DA and MH (red and blue respectively) and that appeared with DA and MH (green and black respectively) and **c** Score plot of PCA of GC–MS data for EOs (green X) and CEEOs (blue circles). The mean value is represented with a +

Given the complexity of the chromatograms of the series of EOs and CEEOs, the GC–MS data were compared using Compass DataAnalysis (DA) and Mass Hunter (MH) software with the aim of finding peaks that were either present in both chromatograms (from EO and CEEO) or found exclusively in one of them. This analysis led to three pools of peaks with a unique combination of m/z and elution time values: (1) peaks found in both chromatograms, likely representing compounds that do not fully react under the applied reaction conditions, (2) peaks exclusively present in the starting EO chromatograms, possibly indicating compounds reacting with NH_4_SCN, and (3) peaks exclusively found in the CEEOs, likely denoting reaction products. Data comparison using DA involved a signal-to-noise ratio (S/N) of five. The average number of peaks per chromatogram at this S/N threshold was similar for EOs and CEEOs (49 and 48, respectively, Fig. 1a). Remarkably, the percentages of peaks that disappeared and appeared were also very similar (Fig. 1b). When comparing GC–MS data with the MH, a filter was applied for peaks with an area equal to or greater than 5% of the main peak (MP). In this case, the average number of peaks per chromatogram was two times higher for CEEOs as compared to the EOs (28 and 14 respectively, Fig. 1a). Nevertheless, the percentages of disappearing and appearing peaks closely mirrored those obtained with the S/N filter in DA. Both analyses revealed that at least 86% of the peaks observed in the EOs chromatograms were consumed during the diversification process, giving rise to products that represented a minimum of 90% of the peaks exclusive to the CEEOs chromatograms (Fig. 1b).

The GC–MS data were also analyzed using the non-supervised method, Principal Component Analysis (PCA). The score plot showed discrimination between two groups by PC1 and PC2. The EOs exhibited positive values for PC2 and negative (or nearly zero) for PC1 placing them in the top left quadrant of the score plot (green x, Fig. 1c) while the CEEOs are distributed in the other quadrants (blue circles, Fig. 1c).

### Alteration of biological properties

The impact of the reaction on the biomolecular properties of the mixtures was evaluated by comparing the AChE inhibitory properties of the CEEOs and EOs. There has been a significant effort to find compounds that could act as AChE inhibitors. This is primarily because there is evidence suggesting that AChE inhibition can be advantageous for Alzheimer’s disease (AD) patients. In AD, the impairment of the cholinergic system plays a role in the decline of cognitive function associated with the disease [58, 59]. Which is why, some reversible inhibitors of this enzyme are the primary class of drugs currently utilized for the symptomatic relief of AD [60].

**Fig. 2:**
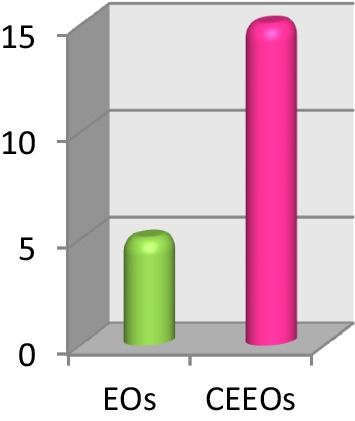
Changes observed in AChE inhibition by the reaction over EOs. **a** Green bars illustrate de biological activity in EOs and pink bars indicate the biological activity present in the CEEOs

The AChE inhibition properties of the mixtures were surveyed by thin layer chromatography (TLC) autography [61], a technique particularly well-suited for the analysis of mixtures [62]. This methodology allows the qualitative evaluation of the inhibitory properties of a sample spotted onto a TLC plate which is then covered with a gel containing enzyme, substrate, and a revealing reagent for the product. When comparing the bioactivity profiles of each CEEO/EO pair, it was observed that the number of inhibition halos increased 3 times because of the modification process (Fig. 2). The alteration of the biomolecular properties of the mixtures from their modification is attractive given that, in addition to expanding the overall number of active mixtures, CEEOs with inhibitory properties against AChE can be generated from previously inactive mixtures (four active CEEOs since eight inactive EOs, Table S1).

### Identification and biological characterization of inhibitors from the CEEO of *O. vulgare*

**Fig. 3:**
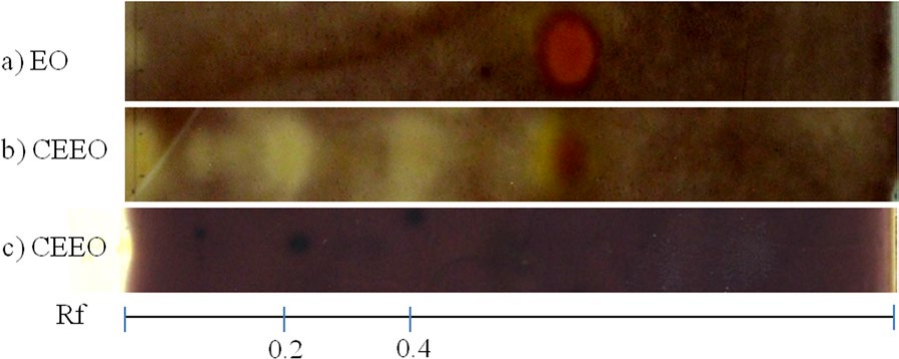
TLC of OV **a** EO revealed with AChE assay, **b** CEEO revealed with AChE assay and **c** CEEO revealed with false-positive AChE assay. Mobile phase hexane: ethyl acetate (75:25)

One of the most interesting findings was in the case of CEEO from OV where the two more intense inhibition zones were detected, at Rf values 0.2 and 0.4, while its parent EO showed no activity (Fig. 3b and a). To differentiate between genuine AChE inhibitors and non-real inhibitors, a comparison was made between a control and the enzymatic assay [61]. Notably, none of the halos detected for the CEEO of OV produced false-positive inhibition spots (Fig. 3c).

An autography-guided chromatography column of the CEEO of OV led to the isolation of four active fractions: F0.2A and F0.2B, which contained the inhibition spot with Rf = 0.2, and F0.4A and F0.4b that contained the inhibition spot with Rf = 0.4. GC–MS analysis revealed that each fraction contained one major compound with the following m/z values: 207.1 in F0.2A, 330.2 in F0.2B, 414.2 in F0.4A, and 446.1 in F0.4B. The identity of the main component of each fraction was established by HRMS and NMR (^1^H and ^13^C) as follows: 5-isopropyl-2-methyl-4-thiocyanatophenol (**1**) in F0.2A, 4,4′-thiobis(5-isopropyl-2-methylphenol) (**2**) in F0.2B, 4-((5-(4-hydroxy-2-isopropyl-5-methylphenyl)-1,2,4-thiadiazol-2-yl)thio)-5-isopropyl-2-methylphenol (**3**) in F0.4A, and 4,4′-((1,2,4- reactions thiadiazole-2,5-diyl)bis(sulfanediyl))bis(5-isopropyl-2-methylphenol) (**4**) in F0.4B. Hence, each inhibition halo detected in the autography of the fractions was generated by two compounds.

**Scheme 1:**
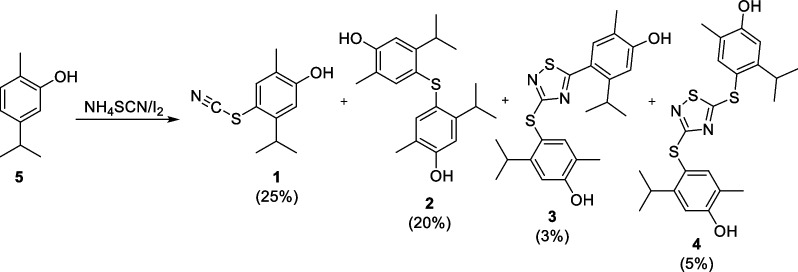
Synthesis of 5-isopropyl-2-methyl-4-thiocyanatophenol (**1**), 4,4′-thiobis(5-isopropyl-2-methylphenol) (**2**), 4-((5-(4-hydroxy-2-isopropyl-5-methylphenyl)-1,2,4-thiadiazol-3-yl)thio)-5-isopropyl-2-methylphenol (**3**), and 4,4′-((1,2,4-thiadiazole-3,5-diyl)bis(sulfanediyl))bis(5-isopropyl-2-methylphenol) (**4**)

The four isolated bioactive compounds shared common chemical features suggesting that their natural precursor could be carvacrol (5, Scheme 1). Carvacrol (Fig S5a), along with its isomer thymol, is a primary constituent of OV EO [63]. Indeed, the EO chromatographic profile obtained by GC–MS supported this observation (Fig S5b). Furthermore, carvacrol was found in significantly lower proportions in the CEEO (Fig S5c). To substantiate this hypothesis, a reaction was carried out using carvacrol under the same reaction conditions that were employed to obtain the CEEO previously. Interestingly, the TLC-AChE inhibition profile of the carvacrol reaction closely resembled that of the CEEO. Subsequently, the four compounds were purified from this mixture and their identities were confirmed through various spectroscopic analyses.

Compounds **1**, **3**, and **4** contain one or two SCN units from the used derivatizing reagent. On the other hand, compound **2** is a thioether. Although the reaction was initially developed to produce thiocyanates or isothiocyanates, recent reports have indicated that reactions with thiocyanate salts can lead to the formation of thioethers [64]. Thioether compounds have become one of the main areas of interest in drug discovery [65]. Besides, in recent years, they acquired more importance in the plant protection field because of their broad spectrum of agricultural activities [66]. This result is interesting since this observed side reaction serves the purpose of diversification of NPs mixtures.

Microplate assays utilizing Ellman’s reagent were performed to quantitatively assess the inhibitory activity of the isolated compounds against AChE [67]. The half maximal inhibitory concentration (IC_50_) values were 4.086 ± 0.035 µM, 0.158 ± 0.059 µM, 0.056 ± 0.025 µM and 0.034 ± 0.016 µM for compounds **1**–**4**, respectively.

While compound **1** demonstrated a reduced inhibitory potency when compared to the reference inhibitor eserine (IC_50_ 0.224 ± 0.017 µM), compounds **2**–**4** exhibited similar or even greater inhibitory activity. Notably, carvacrol was determined to be inactive against this enzyme. This underscores the significance of the reaction in the generating diversity and converting inactive precursors into active compounds that effectively target this biological target.

**Scheme 2:**
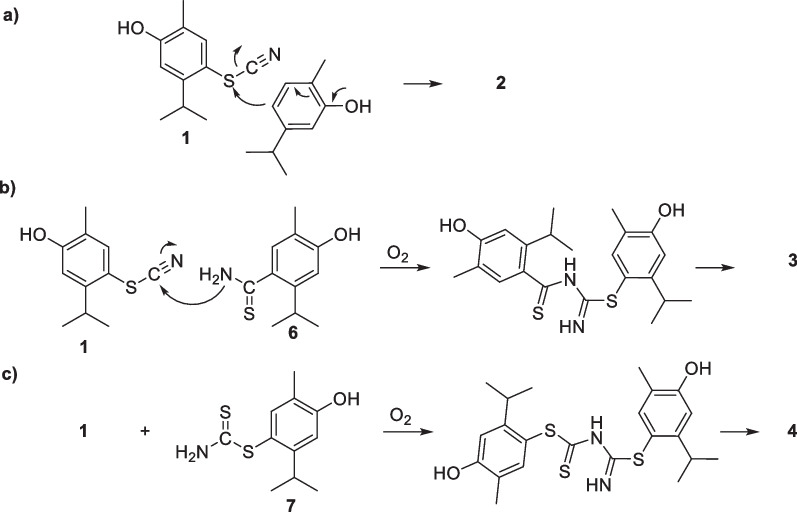
Proposed mechanism for **a** 4,4′-thiobis(5-isopropyl-2-methylphenol) (**2**), **b** 4-((5-(4-hydroxy-2-isopropyl-5-methylphenyl)-1,2,4-thiadiazol-3-yl)thio)-5-isopropyl-2-methylphenol (**3**), and **c** 4,4′-((1,2,4-thiadiazole-3,5-diyl)bis(sulfanediyl))bis(5-isopropyl-2-methylphenol) (**4**)

All the bioactive isolated compounds (**1**–**4**) could be produced by an initial attachment of thiocyanate on the *para* position of the phenol group of carvacrol. The reaction most likely proceeds via the formation of active thiocyanogen and (SCN)_2_ from I_2_ and NH_4_SCN [57, 68]. The *para*-substitution in compound **1** agrees with that observed in aryl thiocyanates obtained by mechanochemical [69], ultrasound [70], K_2_S_2_O_8_ [71]_,_ and CeBr_3_/H_2_O_2_ [72] promoted thiocyanation of C-2 substituted phenols as well as by I_2_ promoted thiocyanation of aromatic amines [55]. The sulfur atom in compound **1** could suffer the nucleophilic attack of an aryl moiety [73–76], here a second carvacrol molecule, leading to the formation of **2** with the release of the cyanide group (Scheme 2a).

1,2,4-thiadiazoles of the type of compounds **3** and** 4** have been conveniently prepared from thioamides or analogous substrates by oxidative heterocyclization [77–80]. Therefore, compound **3** could be achieved by the reaction of **1** with a thioamide such as **6** (Scheme 2a), generated in situ by the reaction of carvacrol with thiocyanate (Scheme 2b) [81]. Similarly, compound **4** could result from an analogous reaction between **1** and dithiocarbamate **7**, generated in situ by the reaction of **1** with thiocyanate (Scheme 2c).

## Conclusions

In summary, this study presents a new approach to create CEEOs through bi-heteroatomic reactions, which, for the first time, utilize a reaction with NH_4_SCN/I_2_ under solvent-free conditions. This methodology offers the opportunity to introduce sulfur and nitrogen atoms into natural compounds in a single step. The robustness of this technique is evident from the changes in the composition of the mixtures, as observed through GC–MS analysis, where most of the EOs components were transformed, expanding the chemical diversity of the mixtures. These modifications in the chemical composition were associated with changes in the inhibition properties against the enzyme AChE. A comparison of the bioactivity profiles of each pair of CEEO/EO revealed a threefold increase in the number of inhibition spots. Notably, in the case of the CEEO of *O. vulgare*, the generation of three active compounds with a similar o higher potency than eserine against enzyme was observed. These results highlight the feasibility of using this reaction as a means to enhance chemical diversity, potentially leading to alterations in biomolecular properties, offering new possibilities for developing new bioactive compounds.

## Experimental

### Chemicals and materials

Ammonium thiocyanate, sodium thiosulfate pentahydrate, anhydrous sodium sulfate and iodine were purchased from Cicarelli Laboratorios (San Lorenzo, Santa Fe). AChE from *Electrophorus electricus* EC 3.1.1.7, Fast blue salt B (FBB), α-naphthol, α-naphthyl acetate (α-NAc), and CDCl_3_, were purchased from Sigma-Aldrich (St. Louis, MO, USA). Acetonitrile (LCMS grade) was acquired from Carlo Erba Reagents (Val de Reuil, France) and formic acid from Merck. Aluminium TLC sheets coated with Silica gel 60 F were purchased from Merck (Darmstadt, Germany). Agar was purchased from Britania (Bs. As., Argentina).

The essential oils (EOs) were purchased from EUMA (Bs. As., Argentina): *Pimenta racemosa* (Mill.) J. W. Moore. (PR), *Thuya occidentalis* L. (TO), *Coriandrum sativum* L. (COS), *Foeniculum vulgare* Mill (FV), *Lavandula angustifolia* Mill. (LA), *Cymbopogon citratus Spreng (*CyC*), Litsea cubeba* (Lour.) Pers. (LC), *Mentha arvensis* L. (MA), *Origanum vulgare* L. (OV), *Pogostemon cablin* Benth. (PC), and *Salvia officinalis* L. (SO).

Gas chromatography mass spectrometry (GCMS) analysis was performed on an Agilent 7890B Gas Chromatograph coupled to Agilent model 5977A Mass Spectrometer. Column: HP-5MS UI, 30 m × 0.25 mm, 0.25 μm film thick. High-resolution mass spectra (HRMS) were recorded on a Q-Exactive (Thermo Fisher Scientific) using a reported method [82].

Nuclear magnetic resonance spectra were recorded on a Bruker Avance II in CDCl_3_ in the presence of TMS (0.00 ppm) as the internal standard (^1^H at 300 MHz and ^13^C at 75 MHz). The assignments were made on the basis of chemical shifts (^1^H and ^13^C), ^1^H multiplicities and using 2D spectrums (COSY ^1^H-^1^H, HSQC and HMBC).

### Preparation of chemically engineered essential oils (CEEOs)

Since the detailed exact composition of the EOs used as starting material is unknown, some average properties of EOs components were used to estimate the appropriate amounts of reagents to be used. The number of moles of reacting molecules was estimated considering 150, as the average molecular weight of EOs components, and one, as the average number of reacting groups per molecule. Finally, two mol of SCNNH_4_ and 0.5 mol of I_2_ per “estimated” mol of EO components were used in the reaction.

A suspension of EO (500 mg), SCNNH_4_ (507.5 mg), and I_2_ (423.1 mg) was stirred during 16 h at ambient temperature. Following, a solution of 15% sodium thiosulfate pentahydrate (60 mL) was added, and the reaction mixture stirred for 5 min. The organic material was then extracted with ethyl acetate (2 × 60 mL), and the organic phase dried over anhydrous sodium sulfate and the ethyl acetate from the joined fractions was removed under reduced pressure. Chemically engineered essential oil yields: PR = 92.5%, TO = 95.0%, COS = 103.9%, FV = 106.9%, LA = 104.0%, CyC = 82.7%, LC = 84.7%, MA = 89.2%, OV = 121.6%, PC = 97.9%, and SO = 92.9%.

### AChE TLC and false positive autographic assays

The AChE inhibition properties of the mixtures were surveyed by TLC autography using reported protocols (Ramallo et al*.*, 2015). Briefly, a Silica gel-TLC plate (80 cm^2^) was sprayed with the α-NAc: FBB (4:1) solution. Then the plate was dried under air current at room temperature. 140 mg of agar was dissolved at 80 °C in 16 mL Tris–HCl buffer (50 mM, pH 8.0), and the solution was allowed to cool down (50 °C) and 136 μL of AChE solution (132 U/mL) were added, the obtained solution was mixed by inversion and distributed over the TLC plate. After cooling and solidification, the plate was incubated at 37 °C (20 min) in the dark (in a closed and humid Petri dish).

For the AChE false positive autographic assay, a Silica gel-TLC plate (80 cm^2^) was sprayed with the α-NAc: FBB (4:1) solution. Then the plate was dried under air current at room temperature. 140 mg of agar was dissolved at 80 °C in 16 mL Tris–HCl buffer (50 mM, pH 8.0), then 600 of ethanolic α-naphthol solution (1.7 mg/mL) were added, the obtained solution was mixed by inversion and finally the agar-α-naphthol solution was distributed over it. The autography was analyzed immediately after it was gelled.

### Fractionation of CEEO of OV

The CEEO of OV (600 mg) was fractionated by column chromatography on Silica gel with hexane: EtOAc gradient: 95:5 (5 dead volumes), 90:10 (2.5 dead volumes), 80:20 (2.5 dead volumes), 70:30 (2.5 dead volumes), 50:50 (2.5 dead volumes), 30:70) (2.5 dead volumes), and pure EtOAc (2.5 dead volumes), giving twenty two fractions. TLC-autography analysis of those fractions detected inhibition halos in four fractions: F0.2A, F0.2B, F0.4A and F0.4B.

GC–MS analysis revealed that each fraction contained one major compound each with the following m/z values: 207.1 in F0.2A, 330.2 in F0.2B, 414.2 in F0.4A, and 446.1 in F0.4B (Fig. S1–S4). The identities of these compounds were established by HRMS and NMR (^1^H and ^13^C) analyses as 5-isopropyl-2-methyl-4-thiocyanatophenol (**1**), 4,4′-thiobis(5-isopropyl-2-methylphenol) (**2**), 4-((5-(4-hydroxy-2-isopropyl-5-methylphenyl)-1,2,4-thiadiazol-3-yl)thio)-5-isopropyl-2-methylphenol (**3**), and 4,4′-((1,2,4-thiadiazole-3,5-diyl)bis(sulfanediyl))bis(5-isopropyl-2-methylphenol (**4**), respectively. The assignments for **1–4** are shown below.

### Synthesis of carvarol derivatives

*Standard procedure* A suspension of carvacrol (250 mg), SCNNH_4_ (253.5 mg), and I_2_ (211.1 mg) was stirred during 16 h at 20 °C. Following, a solution of 15% of sodium thiosulfate pentahydrate (30 mL) was added, and the reaction mixture stirred for 5 min. The organic material was then extracted with ethyl acetate (2 × 30 mL), and the organic phase dried over anhydrous sodium sulfate. Next, ethyl acetate was removed by rotary evaporation, and the crude product was purified by column chromatography over silica gel using hexane: EtOAc gradient: 95:5 (5 dead volumes), 90:10 (2.5 dead volumes), 80:20 (2.5 dead volumes), 70:30 (2.5 dead volumes), 50:50 (2.5 dead volumes), 30:70) (2.5 dead volumes), and pure EtOAc (2.5 dead volumes).

1. Yield: 25%; ^1^H NMR (300 MHz, CDCl_3_) δ = 1.24 (6H, d, *J* = 6.7 Hz, CH(C**H**_**3**_)_2_), 2.22 (3H, s, Ar-C**H**_3_), 3.41 (1H, m, C**H**(CH_3_)_2_), 5.24 (1H, s, OH), 6.77 (1H, s, Ar), 7.41 (1H, s,Ar); ^13^C NMR (75 MHz, CDCl_3_) δ = 15.30 (CH_3_, Ar-**C**H_3_), 23.40 (2 CH_3_, CH(**C**H_3_)_2_), 31.34 (CH, **C**H(CH_3_)_2_), 111.02 (C, Ar), 112.84 (C, Ar), 113.80 (CH, Ar), 124.29 (C, S**C**N), 137.16 (CH, Ar), 150.64 (C, Ar), 157.18 (C, Ar); HRMS: found *m/z* = 206.0642, calculated *m/z* for C_11_H_12_OSN [M-H]^−^ 206.0634 (0.8 mDa error).

2. Yield: 20%; ^1^H NMR (300 MHz, CDCl_3_) δ = 1.20 (12H, d, *J* = 6.8 Hz, 2 CH(C**H**_**3**_)_2_), 2.14 (6H, s, 2 Ar-C**H**_3_), 3.46 (1H, m, 2 C**H**(CH_3_)_2_), 4.67 (1H, s, 2 OH), 6.76 (1H, s, 2 Ar), 6.87 (1H, s, 2 Ar); ^13^CNMR (75 MHz, CDCl_3_) δ = 15.36 (2 CH_3_, Ar-**C**H_3_), 23.65 (4 CH_3_, CH(**C**H_3_)_2_), 30.42 (2 CH, **C**H(CH_3_)_2_), 112.70 (2 CH, Ar), 122.13 (2 C, Ar), 125.36 (2 C, Ar), 134.64 (2 CH, Ar), 148.64 (2 C, Ar), 153.56 (2 C, Ar); HRMS: found *m/z* = 329.1581, calculated *m/z* for C_20_H_25_O_2_S [M-H]^−^ 329.1570 (1.1 mDa error).

3. Yield: 3%; ^1^H NMR (300 MHz, CDCl_3_) δ = 1.19 (6H, d, *J* = 6.7 Hz, CH(C**H**_**3**_)_2_), 1.20 (6H, d, *J* = 6.7 Hz, CH(C**H**_**3**_)_2_), 2.24 (3H, s, Ar-C**H**_3_), 2.26 (3H, s, Ar-C**H**_3_), 3.57 (2H, m, 2 C**H**(CH_3_)_2_), 4.98 (1H, s, OH), 5.14 (1H, s, OH), 6.84 (1H, s, Ar), 6.85 (1H, s, Ar), 7.49 (1H, s, Ar), 7.52 (1H, s, Ar); ^13^CNMR (75 MHz, CDCl_3_) δ = 15.08 (CH_3_, Ar-**C**H_3_), 15.22 (CH_3_, Ar-**C**H_3_), 23.76 (2 CH_3_, CH(**C**H_3_)_2_), 23.79 (2 CH_3_, CH(**C**H_3_)_2_), 29.30 (CH, **C**H(CH_3_)_2_), 31.06 (CH, **C**H(CH_3_)_2_), 112.87 (CH, Ar), 113.15 (CH, Ar), 118.12 (C, Ar), 121.32 (C, Ar), 121.81 (C, Ar), 122.36 (C, Ar), 133.21 (CH, Ar), 139.69 (CH, Ar), 147.83 (C, Ar), 152.36 (C, Ar), 155.95 (C, Ar), 156.64 (C, Ar), 172.16 (C, Ar), 187.96 (C, Ar); HRMS: found *m/z* = 413.1364, calculated *m/z* for C_22_H_25_O_2_N_2_S_2_ [M-H]^−^ 413.1352 (1.2 mDa error).

4. Yield: 5%; ^1^H NMR (300 MHz, CDCl_3_) δ = 1.16 (6H, d, *J* = 6.7 Hz, CH(C**H**_**3**_)_2_), 1.18 (6H, d, *J* = 6.7 Hz, CH(C**H**_**3**_)_2_), 2.19 (3H, s, Ar-C**H**_3_), 2.22 (3H, s, Ar-C**H**_3_), 3.44 (2H, m, 2 C**H**(CH_3_)_2_), 5.07 (1H, s, OH), 5.33 (1H, s, OH), 6.80 (1H, s, Ar), 6.83 (1H, s, Ar), 7.42 (1H, s, Ar), 7.43 (1H, s, Ar); ^13^CNMR (75 MHz, CDCl_3_) δ = 15.15 (CH_3_, Ar-**C**H_3_), 15.23 (CH_3_, Ar-**C**H_3_), 23.75 (2 CH_3_, CH(**C**H_3_)_2_), 23.89 (2 CH_3_, CH(**C**H_3_)_2_), 31.04 (2 CH, **C**H(CH_3_)_2_), 113.26 (CH, Ar), 113.86 (CH, Ar), 116.99 (C, Ar), 117.49 (C, Ar), 122.39 (C, Ar), 123.77 (C, Ar), 139.38 (CH, Ar), 139.52 (CH, Ar), 152.45 (C, Ar), 152.60 (C, Ar), 156.02 (C, Ar), 157.25 (C, Ar), 172.37 (C, Ar), 194.06 (C, Ar); HRMS: found *m/z* = 445.1086, calculated *m/z* for C_22_H_25_O_2_N_2_S_3_ [M-H]^−^ 445.1073 (1.3 mDa error).

### Microplate assays

Inhibition percentage determinations were carried out using the method applied by Osella et al*.* [83]. Wells were filled in triplicate with AChE solution (in 0.1 M phosphate buffer, pH 7.5, and 13.7 µU/mL end concentration per well), Ellman’s reagent (DTNB, 5,5dithio-bis-(2-nitrobenzoic acid)), the same buffer solution (0.31 mM end concentration per well) and 10 µL of test compound in DMSO solution (0.05 µM end concentration per well). Wells containing the corresponding volume of DMSO without an inhibitor were used as references of maximum enzymatic rates, and standard drug eserine was used as the control for enzyme inhibition. The enzymatic reaction was initiated by addition of acetylthiocholine iodide (0.46 mM end concentration per well). The final volume per well was 270 µL. The plate was shaken for 2 s and the increase in absorbance at 405 nm was monitored at 37 °C for 15 min. For IC_50_ determination, ten serial dilutions of the compounds were prepared in DMSO, following equally spaced points on a neperian logarithm scale, starting at 2.16 mM and finishing at 0.00023 mM (end concentration per well: 80.00 to 0.00839 µM). IC_50_ calculated using Prism V5.01 (GraphPad Software Inc., La Jolla, CA, USA) applying a non linear regression curve fit for a log[inhibitor] vs. normalized answer model with variable slope.

### Supplementary Information

Supplementary Material 1.

## Data Availability

The data supporting the results of this study can be obtained from the corresponding authors upon reasonable request.

## Abbreviations

CEEOs: Chemically engineered essential oils
CEEs: Chemically engineered extracts
EOs: Essential oils
GCMS: Gas chromatography mass spectrometry
HRMS: High resolution mass spectrometry
IC_50_: Half maximal inhibitory concentration
NPs: Natural products
NMR: Nuclear magnetic resonance spectroscopy
OV: *Origanum vulgare*
