# Supplementary material for: Chemically engineered essential oils prepared through thiocyanation under solvent-free conditions: chemical and bioactivity alteration
Source: Nat Prod Bioprospect. 2024 Jun 1;14(1):35. doi: 10.1007/s13659-024-00456-w (PMC11143095; doi:10.1007/s13659-024-00456-w)
Supplement: Supplementary file 1 — Supplementary Material 1. [file 13659_2024_456_MOESM1_ESM.docx]

**Electronic Supplementary Material**

**Chemically engineered essential oils prepared through thiocyanation under solvent-free conditions: chemical and bioactivity alteration**

Liz E. Lescano,^a^ Mario O. Salazar ^a,b^ * and Ricardo L. E. Furlan ^a,b^

^a^ Farmacognosia, Facultad de Ciencias Bioquímicas y Farmacéuticas, Universidad Nacional de Rosario (UNR), Suipacha 531, 2000 Rosario, Argentina

^b^ Consejo Nacional de Investigaciones Científicas y Técnicas (CONICET)

E-mail address: msalazar@fbioyf.unr.edu.ar (M.O. Salazar).

**Contents**

**Fig. S1**: GC-MS traces of F0.2A......................................................................................................S2

**Fig. S2**: GC-MS traces of F0.2B......................................................................................................S2

**Fig. S3**: GC-MS traces of F0.4A......................................................................................................S3

**Fig. S4**: GC-MS traces of F0.4B......................................................................................................S3

**Table 1**: Comparing the AChE inhibition properties of each CEEO/EO pair.........................................S4

**Fig. S5**: GC-MS traces of a) carvacrol, b) EO of OV and c) CEEO of OV.......................................S4

Mass spectrum of compounds **1** to **4**...................................................................................................S5

^1^H NMR (300 MHz) of compounds **1** to **4**...........................................................................................S6

^13^C NMR (300 MHz) of compounds **1** to **4**..........................................................................................S8

Plots of % of inhibition of AChE versus log [I] of compounds **1-4** .....................................................S9


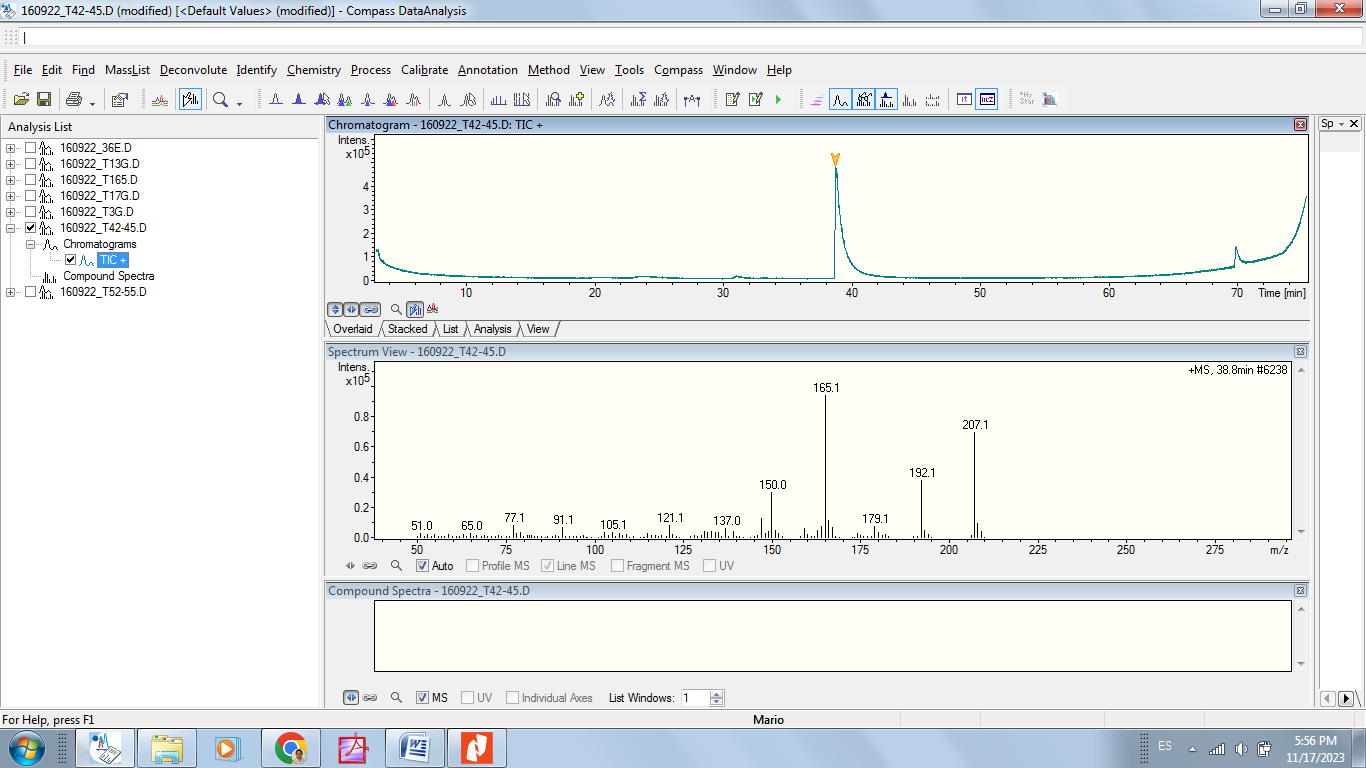


**Fig. S1**: Representative GC- MS traces of F0.2A (up) and mass spectrum of compound at rt 38.8 min (down).


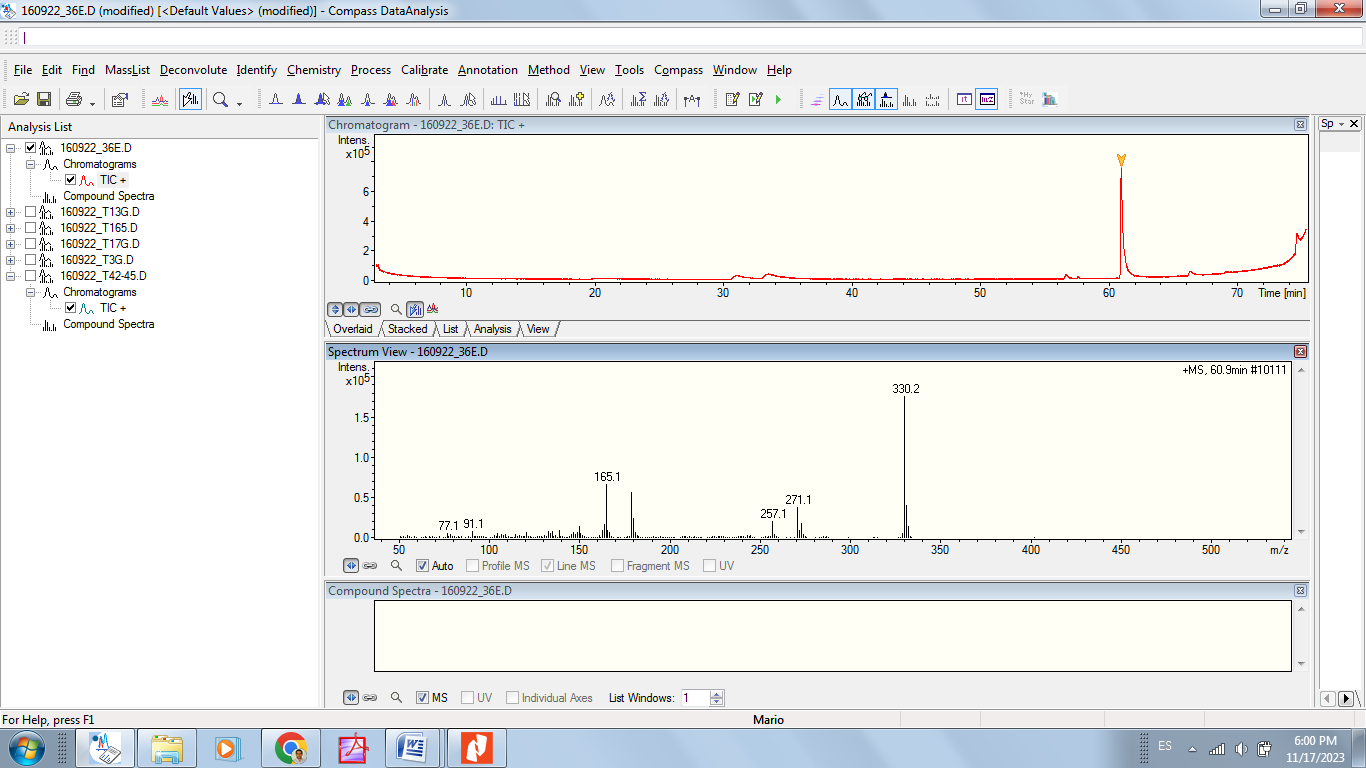


**Fig. S2**: Representative GC- MS traces of F0.2B (up) and mass spectrum of compound at rt 60.9 min (down).


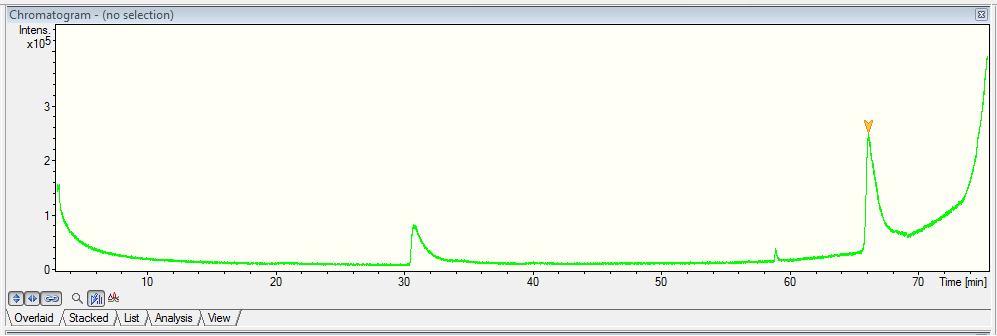

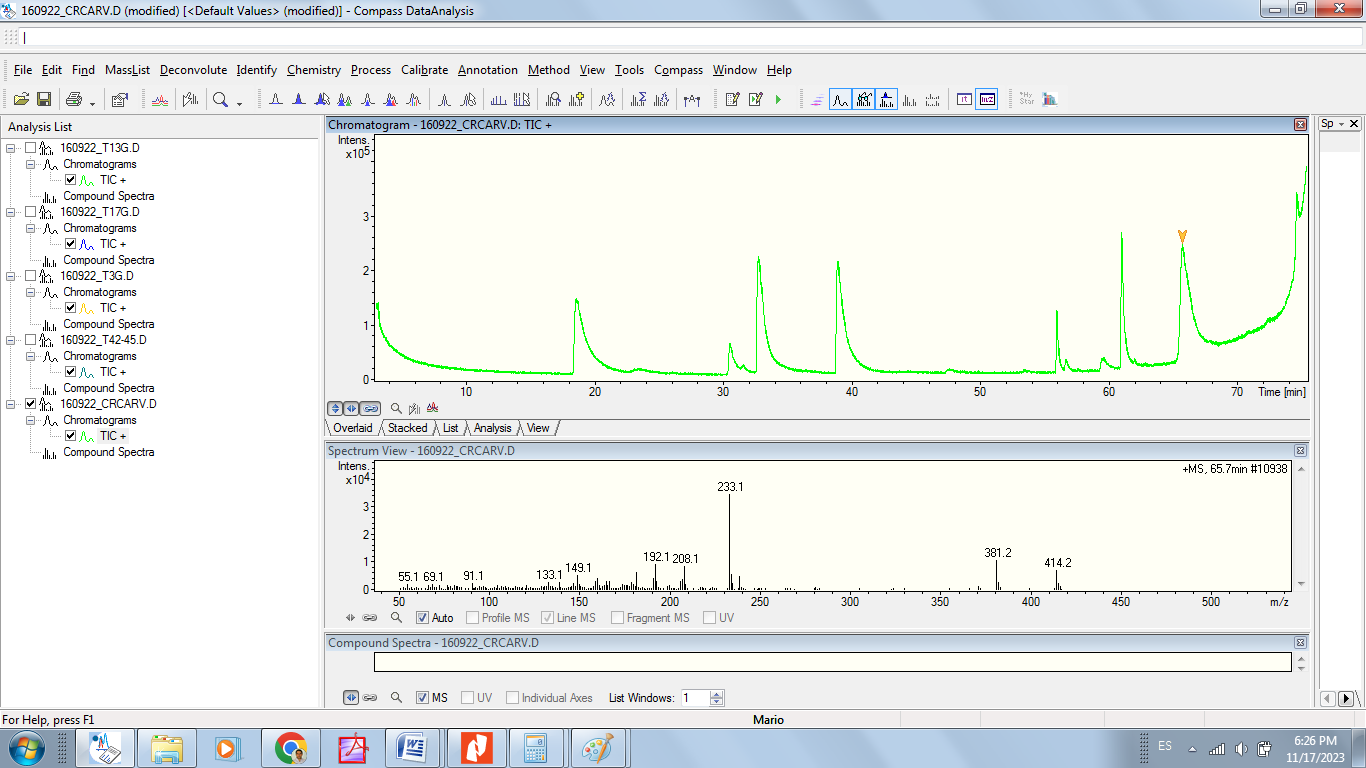


**Fig. S3**: Representative GC- MS traces of F0.4A (up) and mass spectrum of compound at rt 65.7 min (down).


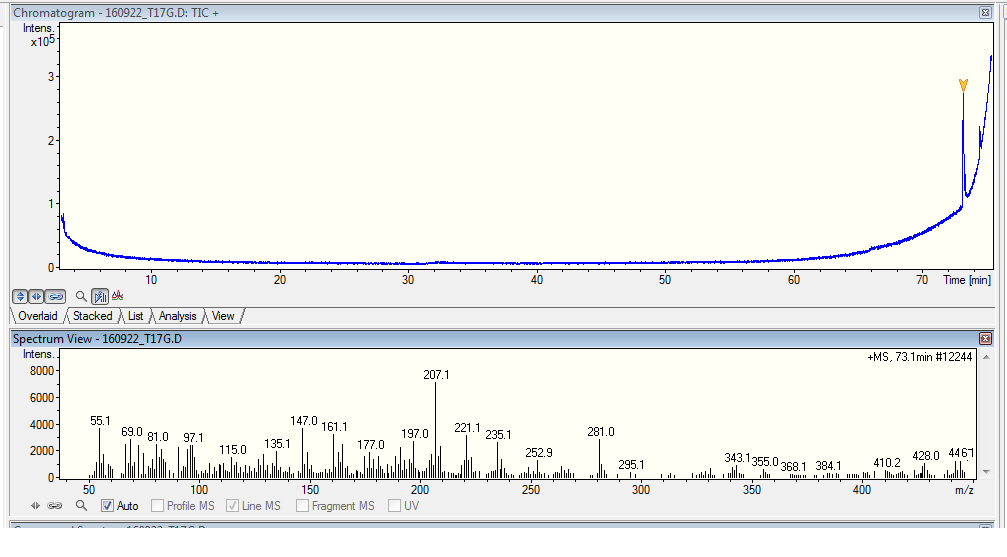


**Fig. S4**: Representative GC- MS traces of F0.4B (up) and mass spectrum of compound at rt 73.1 min (down).

**Table S1.** Comparing the AChE inhibition properties of each CEEO/EO pair.

|  | **Inhibition halos** | | | |
| --- | --- | --- | --- | --- |
| **Samples** | **EOs** | **CEEOs** | **New** | **Total** |
| *Pimenta racemosa* (Mill.) J.W.Moore. | 0 | 0 | 0 | 0 |
| *Thuya occidentalis* L. | 0 | 2 | 2 | 2 |
| *Coriandrum sativum* L. | 2 | 1 | 0 | 2 |
| *Foeniculum vulgare* Mill | 0 | 0 | 0 | 0 |
| *Lavandula angustifolia* Mill. | 0 | 1 | 1 | 1 |
| *Cymbopogom citratus* (DC.) Stapf | 0 | 0 | 0 | 1 |
| *Litsea cubeba* (Lour.) Pers. | 0 | 0 | 0 | 1 |
| *Menta arvensis* L. | 1 | 2 | 1 | 2 |
| *Origanum vulgare* L. | 0 | 2 | 2 | 2 |
| *Pogostemon* cablin Benth. | 2 | 0 | 0 | 2 |
| *Salvia officinalis* L. | 0 | 2 | 2 | 2 |

**Fig. S5**: GC-MS traces of a) carvacrol, b) EO of OV and c) CEEO of OV.

**Mass spectrum**

| **Compound 1**  **** | **Compound 2**  **** |
| --- | --- |
| **Compound 3**  **** | **Compound 4**  **** |

**^1^ H NMR (300 MHz) of compounds 1 to 4**

**Compound 1**

**
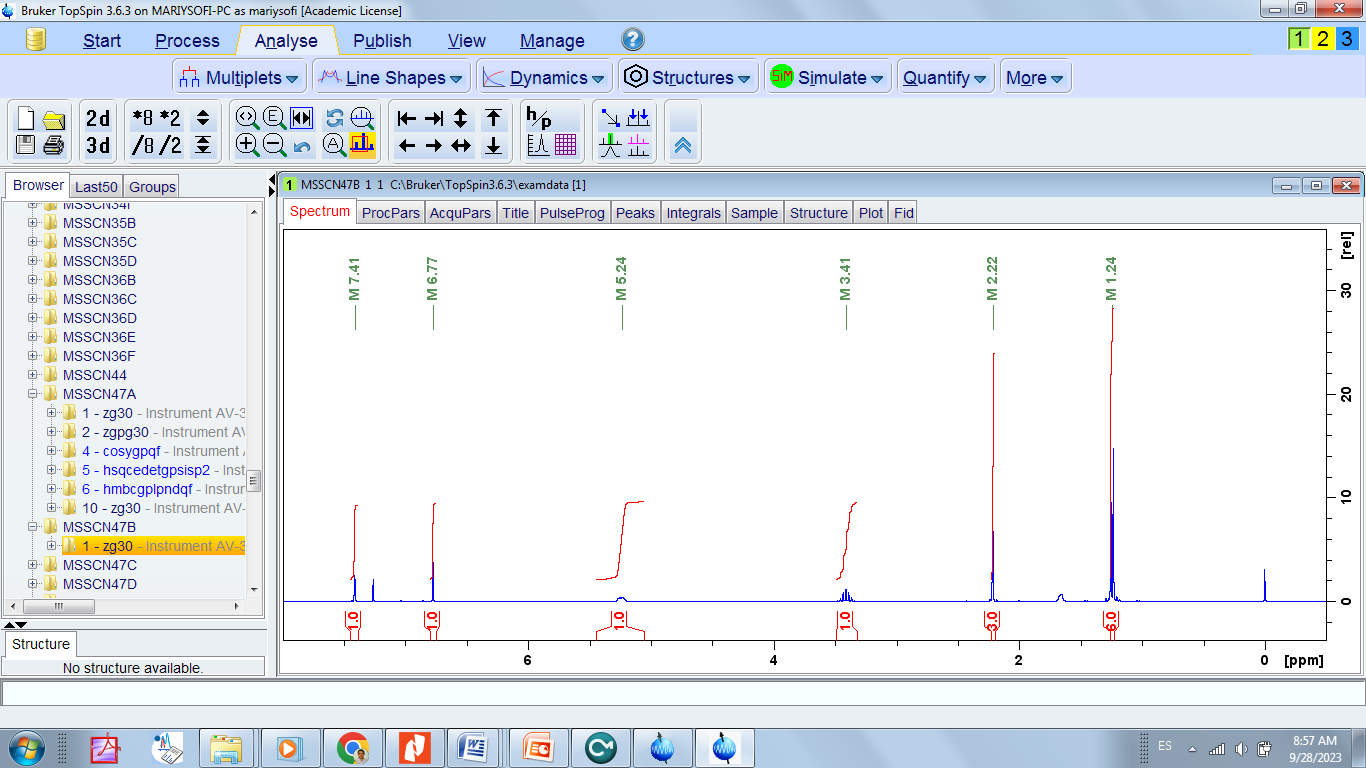
**

**Compound 2**

**
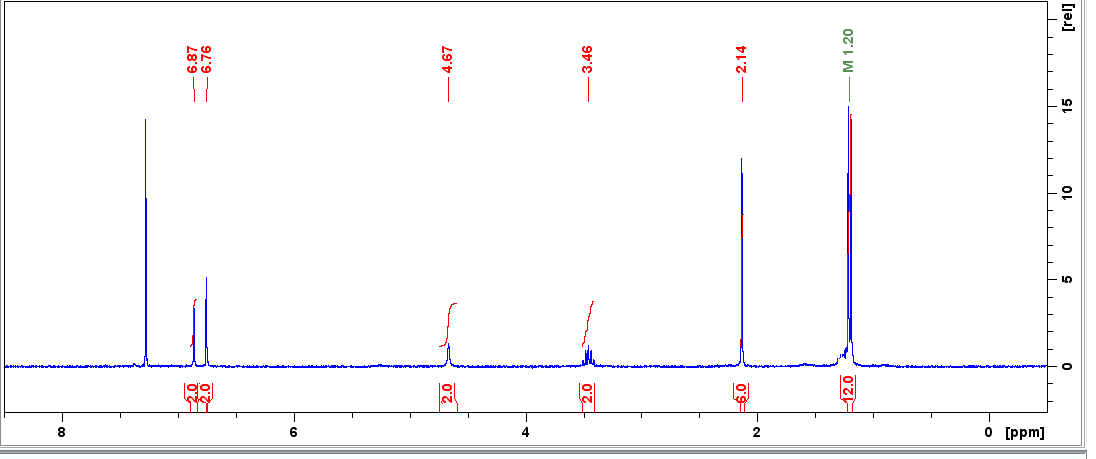
**

**Compound 3**

**
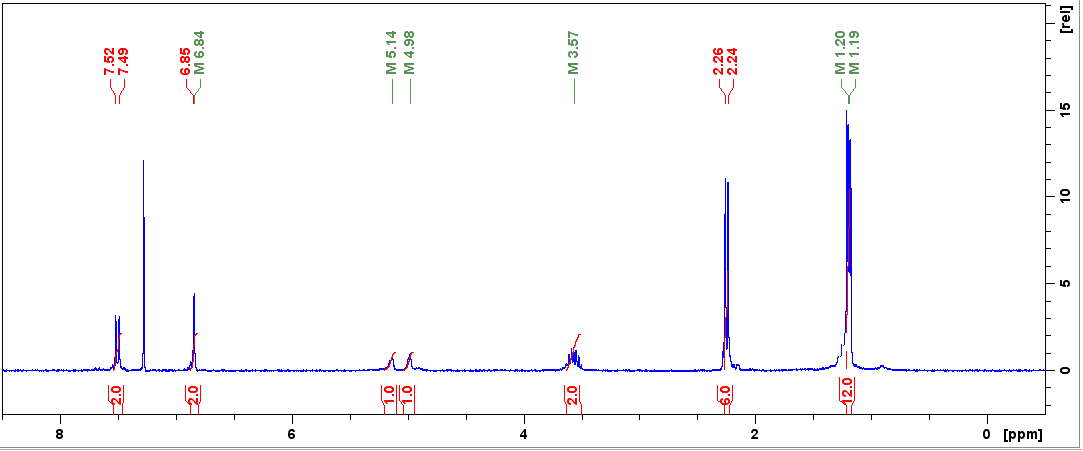
**

**Compound 4**


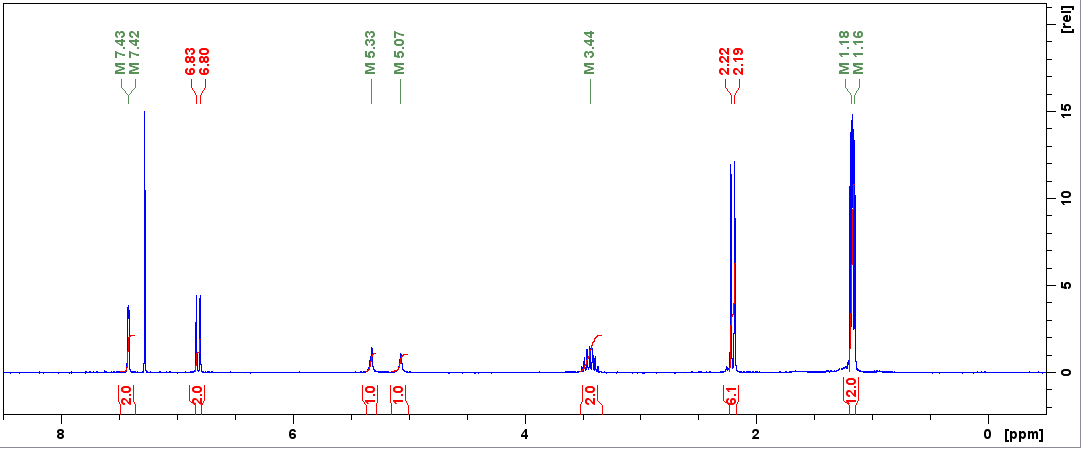


**^1 3^C NMR(75 MHz) of compounds 1 to 4**

**Compound 1**

**
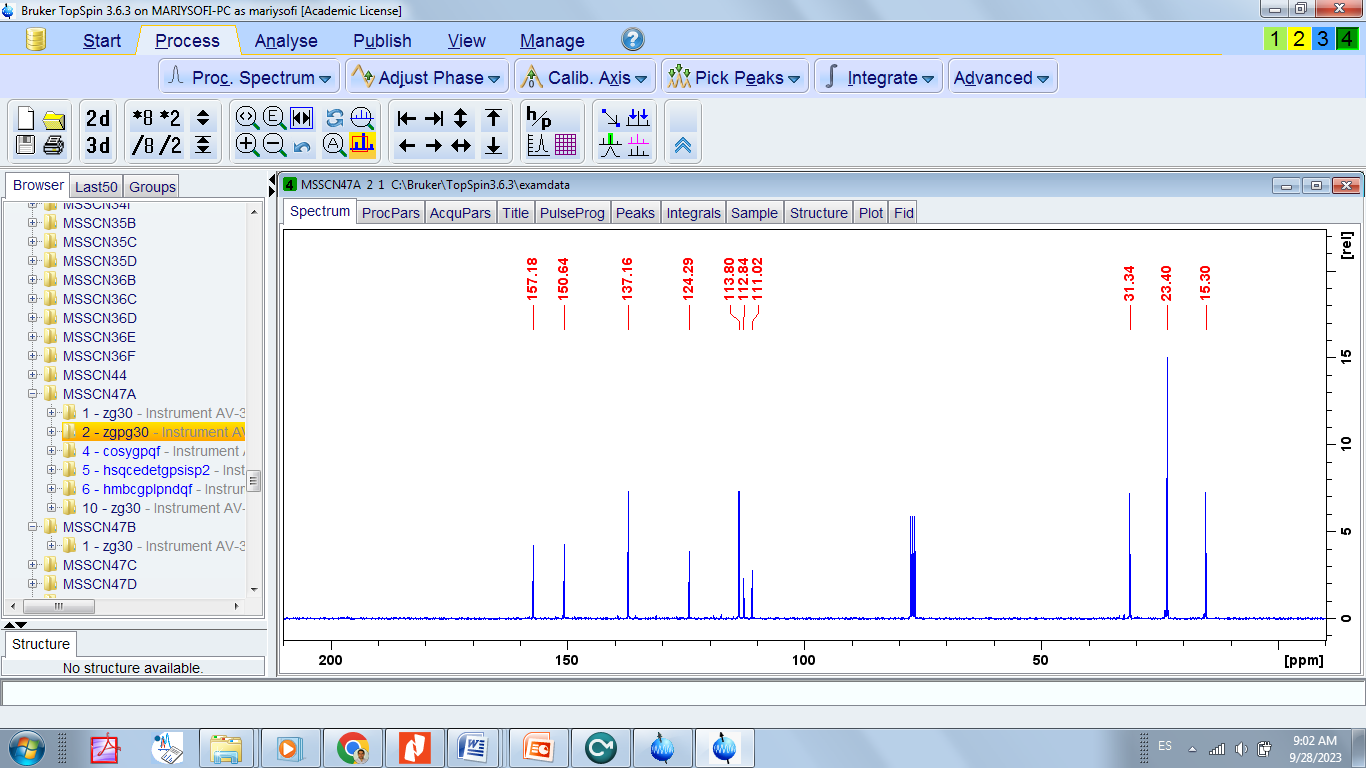
**

**Compound 2**

**
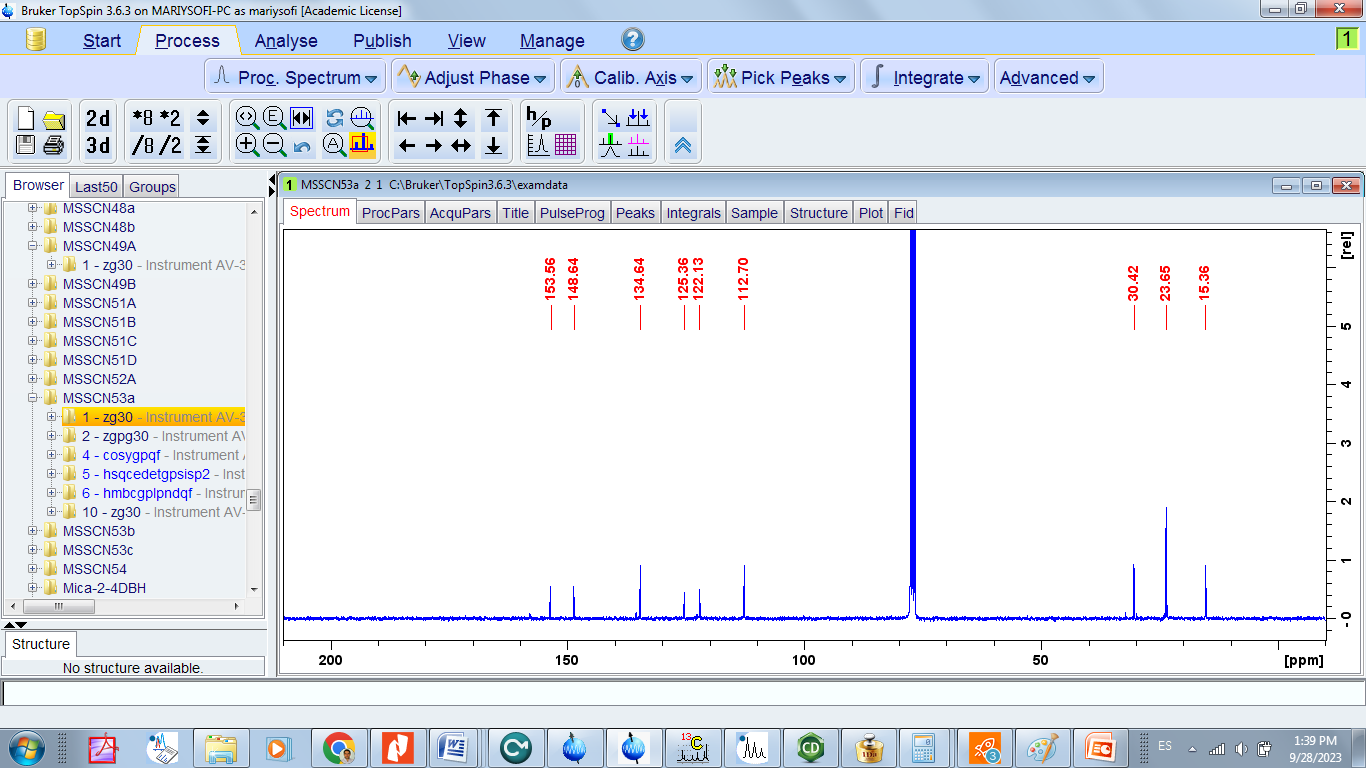
**

**Compound 3**

**
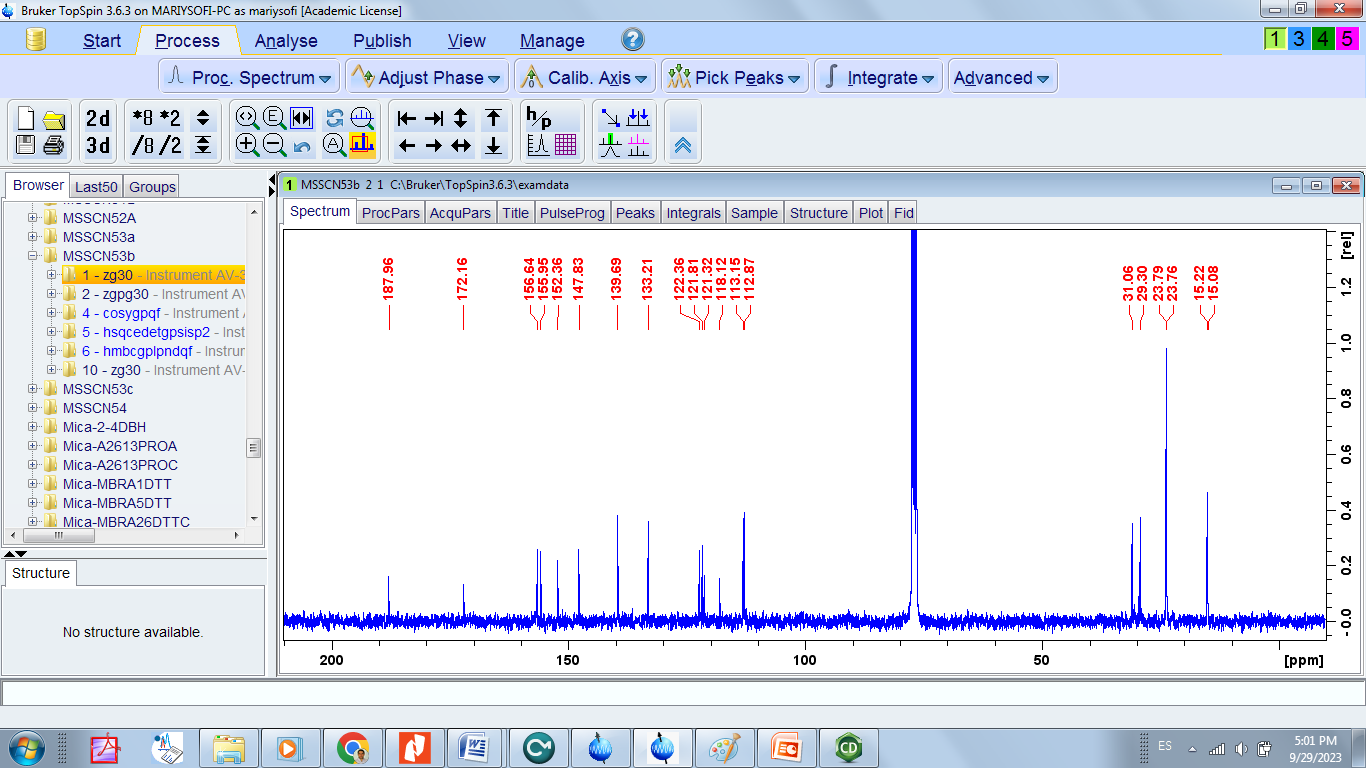
**

**Compound 4**


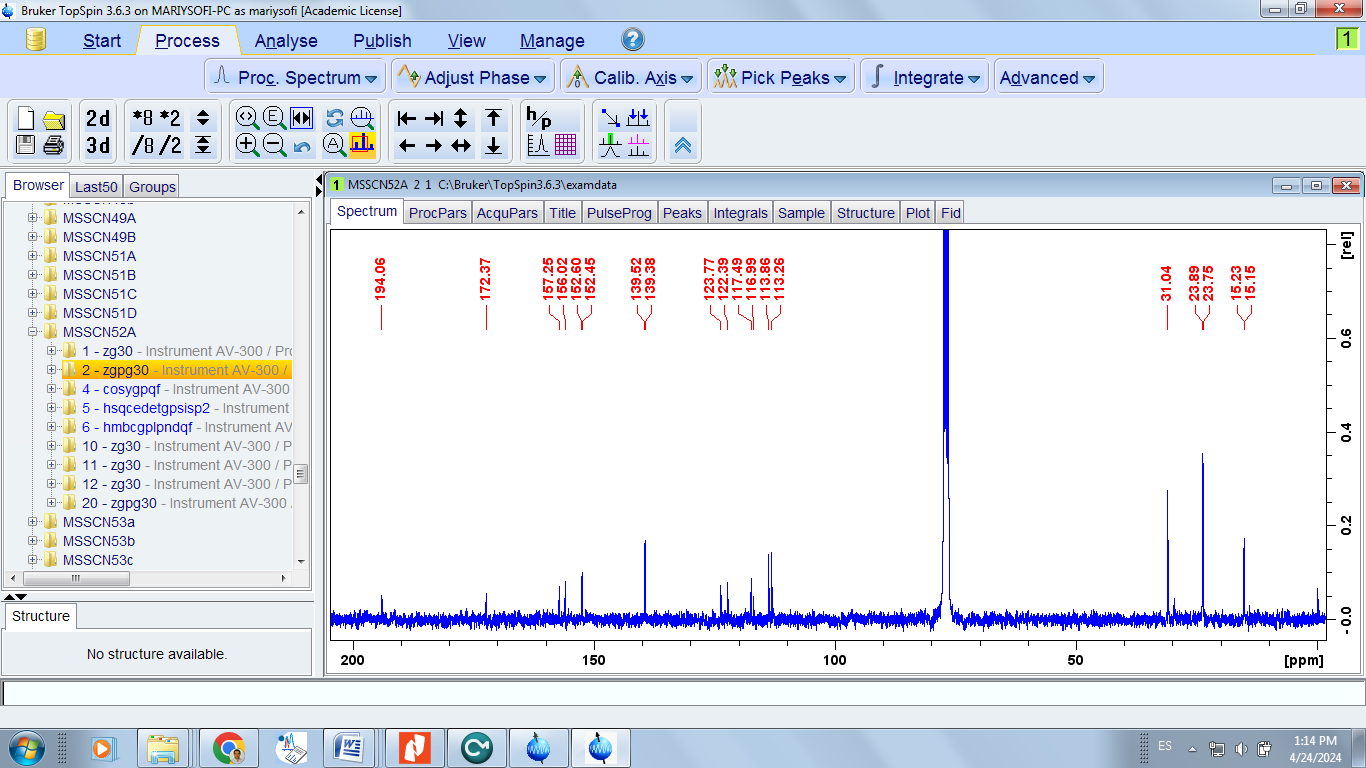


**Plots of % of inhibition of AChE versus log [I] of compounds 1 to 4**

**Compound 1**

**Compound 2**

**Compound 3**

**Compound 4**
